# Cir-ITCH inhibits gastric cancer migration, invasion and proliferation by regulating the Wnt/β-catenin pathway

**DOI:** 10.1038/s41598-020-74452-8

**Published:** 2020-10-15

**Authors:** Yang Peng, Hong Hong Wang

**Affiliations:** 1 Department of Geriatrics, Tongji Hospital, Tongji Medical College, Huazhong University of Science and Technology, Wuhan, 430030 China; 2 Department of Medical Ultrasound, Tongji Hospital, Tongji Medical College, Huazhong University of Science and Technology, 1095# Jiefang Avenue, Wuhan, 430030 China

**Keywords:** Cancer, Gastrointestinal cancer, RNA splicing, RNAi

## Abstract

Circular RNAs (circRNAs) are differentially expressed in various tumours, but the expression and regulatory mechanisms of circular RNA ITCH (cir-ITCH) in gastric cancer remain unclear. For this reason, in the present study, we assessed the expression of cir-ITCH and the associated regulatory mechanism of cir-ITCH in gastric cancer. Through RTq-PCR assays, we observed that cir-ITCH expression was attenuated in gastric cancer cell lines and tissues, with cir-ITCH expression in gastric cancer tissues with lymph node metastasis being considerably lower than that observed in gastric cancer tissues without lymph node metastasis. In addition, we demonstrated that cir-ITCH or linear ITCH may be a useful marker for gastric cancer prognosis by Kaplan–Meier survival analysis. We also showed that cir-ITCH overexpression could increase linear ITCH expression through miR-17 via RNA immunoprecipitation (RIP) and luciferase reporter assays. Moreover, in vivo and in vitro experimental results showed that cir-ITCH can act as a tumour suppressor to prevent gastric cancer tumourgenesis by sponging miR-17. The Wnt/β-catenin pathway plays a crucial role during the carcinogenesis of cancers, and we observed that cir-ITCH could negatively regulate Wnt/β-catenin signalling, which could be restored by miR-17. In summary, cir-ITCH was shown to prevent gastric cancer tumourgenesis through the Wnt/β-catenin signalling pathway by sequestering miR-17.

## Introduction

Gastric cancer is one of the most common human cancers^1^. Recent studies have shown that gastric cancer, for which there were 1,000,000 newly diagnosed cases and 783,000 deaths in 2018, remains the 5th most diagnosed cancer and the 3rd leading cause of cancer-related death^2^ . The formation of human gastric cancer is thought to be greatly affected by genetic and epigenetic mutations, including the overexpression of oncogenes and the downregulation of tumour suppressor genes^3^. miRNAs can regulate the expression of tumour suppressor genes and oncogenes by binding to the 3′UTRs of mRNA, and recent studies having shown that circular RNAs (circRNAs) possess miRNA binding sites and function as sponges to sequester miRNAs^4,5^.

circRNAs are a distinct class of endogenous noncoding RNAs that are formed by back-splicing events via exon or intron circularization^6,7^. Although circRNAs were first detected decades ago, their functions in mammalian cells have only recently been elucidated. circRNAs are widely involved in the formation of RNA–protein complexes, act as miRNA sponges, and regulate targeted gene transcription and splicing^8–10^. Many studies have demonstrated the crucial roles of circRNAs in the development of tumours. For example, Mantang et al. showed that the circular RNA circPRKCI promotes tumour growth in lung adenocarcinoma^11^, while William W et al. observed that Foxo3 circular RNA retards cell cycle progression by forming ternary complexes with p21 and CDK2^12^.

The E3 protein ubiquitin ligase (E3) ITCH was originally identified through genetic studies investigating the agouti locus, mutations in which result in coat colour alterations in mice^13^. ITCH harbours 4WW domains and can bind to PPxY-containing targets^14^, with the downstream genes of ITCH having highly important roles in chemosensitivity and tumour formation^15^. Cir-ITCH is formed by noncanonical splicing of the ITCH gene, which is located on chromosome 20q11.22 on the plus strand and spans exons 6–13. Cir-ITCH can inhibit lung cancer cell proliferation by disrupting the Wnt/β-Catenin pathway signalling^16^. In ESCC (oesophageal squamous cell carcinoma), cir-ITCH can increase ITCH expression by sponging miR-7, miR-14 and miR-214, and elevated ITCH levels inhibit ESCC tumourigenesis by suppressing the Wnt/β-Catenin pathway^17^. Wnt/β-catenin pathway can also be regulated by cir-ITCH in colorectal cancer by sponging miR-7 and miR-20a^18^. In addition to regulating the Wnt/β-catenin pathway, cir-ITCH can also affect the expression of other genes. In bladder cancer, cir-ITCH can regulate p21 and PTEN expression by sponging miR-17 and miR-224, and the resulting p21 and PTEN overexpression can suppress the aggressive biological behaviours of bladder cancer^19^. However, no studies have investigated the functional roles of circRNAs in gastric cancer.

In our present study, we assessed the expression of cir-ITCH in gastric cancer and examined the prognostic potential of cir-ITCH in gastric cancer. Furthermore, we investigated the interaction between cir-ITCH, linear ITCH and miRNA and evaluated the roles of cir-ITCH and its downstream genes in vitro and vivo.

## Materials and methods

### Statements, human tissue specimens and cell lines

All experiments were performed in accordance with relevant guidelines and regulations. Fifty human gastric cancer samples and their adjacent normal tissues were obtained from patients who underwent surgery at the Department of Surgery of Tongji Hospital of Tongji Medical College between 2014 and 2015. We received approval for this study from the Institutional Review Board of Tongji Medical College of Huazhong University of Science and Technology, and all patients provided signed informed consent forms.

The normal gastric mucosal cell line GES-1 from the Type Culture Collection of the Chinese Academy of Sciences (Shanghai, China) and gastric cancer cell lines from the American Type Culture Collection (ATCC; Manassas, VA, USA) were cultured in RPMI1640 (HyClone, Logan, UT, USA) supplemented with 10% foetal bovine serum (FBS; Invitrogen, Carlsbad, CA, USA) at 37 °C under a humidified atmosphere with 5% CO_2_. The three 3 cell lines were authenticated by STR profiling and tested for mycoplasma contamination.

### Oligonucleotide transfection and circular RNA plasmid construction

Both miRNA mimics and cont-miR were purchased from Sangon Biotechnology (Sangon, Shanghai, China) and transfected into cells using Lipofectamine 2000 (Invitrogen, Camarillo, CA, USA). Human cir-ITCH cDNA was PCR amplified from AGS cell gDNA, and the PCR product was purified with a miRNAVana miRNA kit (Life Technologies, New York, USA). Finally, the cir-ITCH cDNA was cloned into pCDNA3.1 (Clontech Laboratories, Inc., San Francisco, CA, USA) as previously described^4^. The recombinant plasmid harbouring cir-ITCH was then verified by sequencing, and the lentivirus was constructed by Geneseed (Guangzhou, China). The human miR-17 gene was also PCR amplified and then cloned into a lentiviral vector according to the manufacturer’s instructions^20^.

### RNA extraction and quantitative real-time polymerase chain reaction (RT-qPCR)

Total miRNA was extracted with RNAiso for Small RNA (TaKaRa Bio, Otsu, Japan) following the manufacturer’s instructions. Total RNA was extracted from human tissue specimens and cultured cells using TRIzol reagent (TaKaRa Bio, Otsu, Japan) according to the protocol. Poly-A tails were added to miRNA and U6 using miRNA reaction buffer mixture (TaKaRa Bio, Otsu, Japan), after which total miRNA was reverse transcribed into cDNA using miRNA PrimeScript RT enzyme Mix (TaKaRa Bio, Otsu, Japan). cDNA was synthesized from total RNA using SuperScript III (Invitrogen). RT-qPCR was performed in a CFX96 Real-Time PCR Detection System (Bio-Rad, USA). TaqMan-based RT-qPCR was performed to assess the expression of cir-ITCH in gastric cancer tissues, while SYBR Green was used for RT-qPCR to assess the expression of cir-ITCH, linear ITCH, miRNAs and other genes in gastric cell lines. Primers were synthesized by Sango Biotech, China (Table 1). U6 and GAPDH were used as an internal reference for miRNA, mRNA and circRNA detection. All reactions were performed in triplicate.

**Table 1:** Primers sequences used for qRT-PCR.

Gene	Forward primer	Reverse primer	Probe
cir-ITCH (Taqman)	GCAGAGGCCAACACTGGAA	TCCTTGAAGCTGACTACGCTGAG	CCGTCCGGAACTATGAACAACAATGGCA
GAPDH (Taqman)	CCATGACCCCTTCATTGACC	TTGATTTTGGAGGGATCTCG	CTGAGAACGGGAAGCTTGTC
Linear ITCH	TAGACCAGAACCTCTACCTCCTG	TTAAACTGCTGCATTGCTCCTTG	
Linear ITCH1	GGAGACAACGCCTTAACC	CATCTACTGTGACCTCTACG	
Linear ITCH2	CAACAGAGACAATAGGAGACT	TCAGAAGTGGCAGATGGT	
Linear ITCH3	GATAACATCCAGTAACCACAG	ATAACAGCCACCTTGACAG	
Linear ITCH4	GGCAGCACTGGATTGTATA	GCAACCACTGAAGGAACT	
Circular ITCH	ACAAGAACAACAACGTGGCA	GCTCTTTGTCACCTCCAAGC	
c-Myc	TTCGGGTAGTGGAAAACCAG	CAGCAGCTCGAATTTCTTCC	
CyclinD1	GAGGAGCAGCTCGCCAA	CTGTCAAGGTCCGGCCAGCG	
GAPDH	GAAGGTGAAGGTCGGAGTC	GAAGATGGTGATGGGATTTC	
miR-216b	ACACACTTACCCGTAGAGATTC	Universal primer	
miR-17	CAAAGTGCTTACAGTGCAGGTA	Universal primer	
miR-214	ACAGCAGGCACAGACAGGCAGT	Universal primer	
miR-7	TGGAAGACTAGTGATTTTGTT	Universal primer	
miR-128	TCACAGTGAACCGGTCTCTTT	Universal primer	
U6	GGGCAGGAAGAGGGCCTAT	Universal primer	

### Nucleic acid electrophoresis

The cDNA and gDNA PCR products of cir-ITCH were detected via 2% agarose gel electrophoresis at 100 V for 30 min. The DNA marker used was D0107 (Beyotime, China), and the results were visualized by UV irradiation.

### RNase R digestion

Total RNA (4 µg) was incubated for 30 min at 37 °C with 10 × reaction buffer (20 mM Tris–HCl (pH = 8.0), 0.1 M KCl and 0.1 mM MgCl_2_) and 12 U of RNase R (Epicentre Biotechnologies, Madison, WI, USA), after which total RNA was purified by ethanol precipitation^21^.

### RNA immunoprecipitation (RIP)

We used an EZMagna RIP kit (Millipore, Billerica, MA, USA) to perform RIP according to the manufacturer’s protocols. The gastric cancer cells lines MKN45 and AGS were lysed using RIPA lysis buffer, and then lysates were incubated with magnetic beads conjugated with different antibodies (Ago2 or IgG; Millipore) for 6 h at 4 °C. Then, the beads were rinsed and incubated with Proteinase K to remove the proteins. Subsequently, cir-ITCH expression was assessed by RT-qPCR.

### Biotin-coupled miRNA capture

We transfected biotin-coupled miRNA mimics and control miRNA (Sangon, Shanghai, China) into AGS cells using Lipofectamine 2000 (Invitrogen, Camarillo, CA, USA) and harvested cells after 24 h. Subsequently, the AGS cells were washed twice with PBS and then lysed with lysis buffer. Then, 50 µl of the washed streptavidin magnetic bead samples were blocked for 2 h before being added to tubes to pull down the RNA complexes. Subsequently, the tubes were put on a rotator at low speed (10 r/min) for 4 h and then rinsed five times with lysis buffer. Finally, we used TRIzol LS (Life Technology, USA) to recover RNA, and cir-ITCH expression was assessed by RT-qPCR analysis.

### Luciferase reporter assay

Wild-type cir-ITCH sequences were RT-PCR amplified, and then the sequences were inserted into the pmirGLO luciferase vector (Geneseed, Guangzhou, China). The Mutant cir-ITCH sequences were synthesized using a site-directed Gene Mutagenesis Kit (Beyotime, China) following the manufacturer’s protocol. The linear ITCH vector harbouring the wild-type or mutant 3′UTR region that can bind to miR-17, were also constructed by using a similar method. The vector and miR-17/214 mimics were cotransfected into AGS gastric cancer cells using Lipofectamine 2000. Finally, we examined the luciferase activity of cells using a dual-luciferase reporter assay system (Promega) 48 h after transfection. The relative luciferase activity was normalized to the Renilla luciferase internal control. The primers used to construct the luciferase vector are shown in Table 2.

**Table 2:** Primers sequences used for constructing luciferase vector.

Vector	Forward primer	Reverse primer
Wild type of cir-ITCH	CCGCTCGAG-TTGAAGAAGTAGTTGTGACTTTGC	ATTTGCGGCCGC-CCCATCCAGGTGGCAATGGACCAA
Mutant cir-ITCH for miR-17	AATGGTGAAA-gacatca-TTCAGAAAGTG	CACTTTCTGAA-tgatgtc-TTTCACCATT
Mutant circITCH for miR-214	CCTCTTA-gactacctgc-AGGCTCAGGCCCTAG	CTAGGGCCTGAGCCT-gcaggtagtc-TAAGAGG
Wild type 3′UTR of linear ITCH	CCGCTCGAG-CTTCTGAGAACTTGCACCATGAAT	ATTTGCGGCCGC-GAATCACTTATGTCTTTAGAT
Mutant type 3′UTR of linear ITCH	TTATTAACTGATTAAT-tagcagta-AAGTTCTCTGG	CCAGAGAACTT-tactgcta-ATTAATCAGTTAATAA

The TCF-LEF reporter was purchased from SABiosciences (Qiagen, Inc., Valencia, CA, USA), and we examined reporter activity using a dual-luciferase reporter assay following the manufacturer’s protocol^22^.

### Antibodies and immunoblotting

Antibodies against ITCH (#ab220637), β-catenin (#ab16051), Wnt3a (#ab219412), β-actin (#ab179467), phospho-Dvl (#ab124933) and Dvl (#ab233003) were purchased from Abcam (Cambridge, UK). HRP-conjugated goat anti-rabbit IgG (#sc-2004) was purchased from Santa Cruz Biotechnology. Gastric cancer cells and gastric cancer tissues were lysed using RIPA lysis buffer (Beyotime, China) following the manufacturer’s protocols. Subsequently, the proteins in samples were separated via 10% SDS polyacrylamide gel electrophoresis and then transferred onto PVDF membranes. The membranes containing total proteins were blocked in 5% non-fat milk in TBST for 1 h and then incubated with primary antibodies overnight. Then, we washed the membranes with TBST 3 times and incubated them with HRP-conjugated secondary antibodies for 1 h. Finally, the protein bands were detected using enhanced chemiluminescence reagent (Millipore, Billerica, MA, USA).

### Cell viability and clonability assays

The transfected cells were seeded into 96-well plates at a density of 1 × 10^4^ cells/well, and cell viability was measured using the cell counting kit-8 (CCK-8) system (Dojindo, Japan) following the manufacturer’s protocols. CCK-8 solution (10 μl) was added to each well, and the plate was incubated at 37 °C for 1 h. Then, the absorbance of each well at 450 nm was measured using a microplate reader (Tecan, Switzerland). For the colony formation assay, cells were cultured at a low density (1000 cells/plate) for approximately 2 weeks, after which they were stained with Giemsa, and the colonies were counted.

### Migration and invasion assays

For migration assays, 1 × 10^4^ cells were transferred to a transwell insert with 8-µm pores (BD Biosciences). For invasion assays, 2 × 10^5^ cells were transferred to the top chamber of a transwell with a Matrigel-coated membrane (BD Biosciences). Then, medium supplemented with 10% serum was added to the lower chamber, and cells were cultured in serum-free medium in the top chamber. After incubating the cells in a tissue culture incubator for 16 h, we used cotton-tipped swabs to remove the cells from the upper sides of the membrane. Finally, we stained the migrated/invaded cells with crystal violet and counted the cells.

### In vivo tumour growth assays

Male athymic nude mice were purchased from the Animal Experimental Center of Tongji Hospital of Tongji Medical College and were acclimated for 2 weeks before being injected with AGS gastric cancer cells. This experiment was undertaken according to the guidelines for the Care and Use of Laboratory Animals of Tongji Medical College. All procedures were approved by the Committee on the Ethics of Animal Experiments of Tongji Hospital of Tongji Medical College. We used sodium pentobarbital anaesthesia to minimize the suffering of nude mice throughout the assay. During the experiment, the investigator was blinded to the group allocation. Male nude mice were randomly divided into 3 groups (n = 3). Equal numbers of AGS cells (10^6^) overexpressing cir-ITCH and with or without miR-17 restoration were suspended in 100 μl of PBS and subcutaneously injected into the right rear flank of each mouse. We used the formula V = 1/2 a × b^2^ (a: longest tumour axis; b: shortest tumour axis) to calculate the tumour volume. During the animal experiments, if nude mice died or the xenograft tumour did not grow, we excluded those mice from subsequent experiments. After 5 weeks, all nude mice were sacrificed, and the tumours were excised and preserved in formalin or liquid nitrogen.

### Tumour engraftment and PDTX maintenance

We chose one primary gastric cancer sample and cut it into fragments (1 mm^3^) in Matrigel Basement Membrane Matrix (0.1 ml, 50%, BD Biosciences). Then, the fragments were implanted into the dorsal flank of nude mice (n = 5). Nude mice with palpable tumours were divided into 2 groups, the 1.5 mg/kg empty vector group or the cir-ITCH vector group, with mice in each group receiving an intratumoural injection twice a week for 2 weeks. After 2 weeks, all nude mice were sacrificed, and the tumours were weighed and preserved in formalin or liquid nitrogen.

### Immunohistochemistry

Paraffin-embedded blocks were sectioned onto positively charged microscope slides and then were deparaffinized with xylene. After deparaffinization, the slides were hydrated with absolute ethanol and pretreated with citrate buffer for 20 min in a 98 °C steamer to retrieve the antigens. Then, the slides were incubated with antibodies against ITCH (1:200, Abcam, Cambridge, UK) or β-catenin (1:250, Abcam, Cambridge, UK) at 4 °C overnight. Subsequently, an UltraSensitive S-P Detection kit (KIT-9720, Maixin, Fuzhou, China) was used to perform immunostaining, and a DAB kit (PW017, Sangon Biotech, Shanghai, China) was used to develop colour. Finally, the samples were counterstained with haematoxylin, after which the integrated optical density (IOD/Area) in different groups were examined using Image-Pro Plus 6.0.

### Statistical analysis

The data are presented as the means ± standard error of the mean (s.d.). Difference between two groups were analysed by Student’s t-test. Spearman’s rank test was used to evaluate the relationships between the relative expressions of cir-ITCH and linear ITCH in gastric cancer tissues. Kaplan–Meier (log-rank test) analysis was performed to calculate overall survival time. Differences were considered significant at *p* < 0.05.

## Results

### Identification and characterization of cir-ITCH in gastric cancer

**Figure 1:**
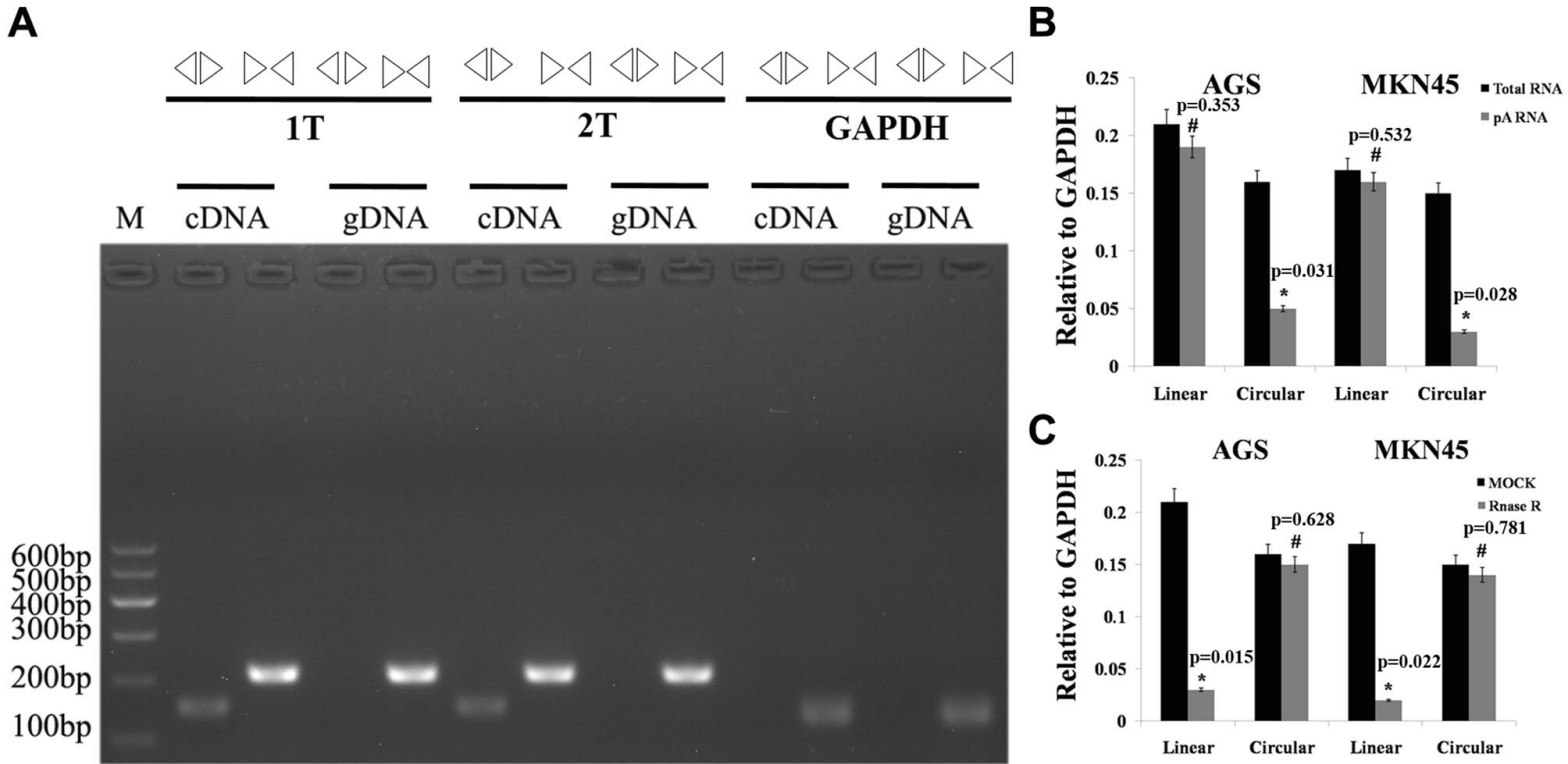
The association between cir-ITCH and gastric cancer. (**A**) Both linear and circular RNAs could be amplified by convergent primers, whereas only circular RNAs could be amplified by divergent primers. Circular RNAs could be amplified from cDNA but not genomic DNA (gDNA). GAPDH was used as a linear control. (**B**) In the reverse transcription experiments, both random primers and oligo(dT) primers were used. The predicted circular RNA was not detected using oligo(dT). (**C**) Cir-ITCH was resistant to RNase R treatment. The data are presented as the means ± s.d. (n = 3) for the cell lines *, *p* < 0.05.

We designed convergent and divergent primers to detect linear and circular RNA expression, respectively. cir-ITCH could be amplified from cDNA but not gDNA using the divergent primers, while linear ITCH could be amplified from both the gDNA and cDNA templates (Fig. 1A). To further characterize cir-ITCH, we constructed a cir-ITCH overexpression vector as described in a previous study^18,23^ The constructed vector was transfected into the gastric cancer cell lines AGS and MKN45 using lipofectamine 2000. Then, random and oligo (dT) primers were used to reverse transcribe total RNA and mRNA into cDNA, respectively. We inferred that cir-ITCH could not be detected in the poly-(A)-enriched samples, in contrast to linear ITCH. We observed that linear ITCH expression (normalized to GAPDH) did not differ between the total RNA and poly(A)-enriched RNA samples in both AGS and MKN45 cells, whereas cir-ITCH expression was attenuated in the poly(A)-enriched RNA compared with that observed in total RNA in these two gastric cancer cell lines (Fig. 1B). RNase R is a 3′ to 5′ exoribonuclease that cannot affect circular RNA but degrades linear RNA^24,25^. As expected, cir-ITCH but not linear ITCH was resistant to RNase R treatment (Fig. 1C).

### Cir-ITCH expression in gastric cancer cell lines and tissues

**Figure 2:**
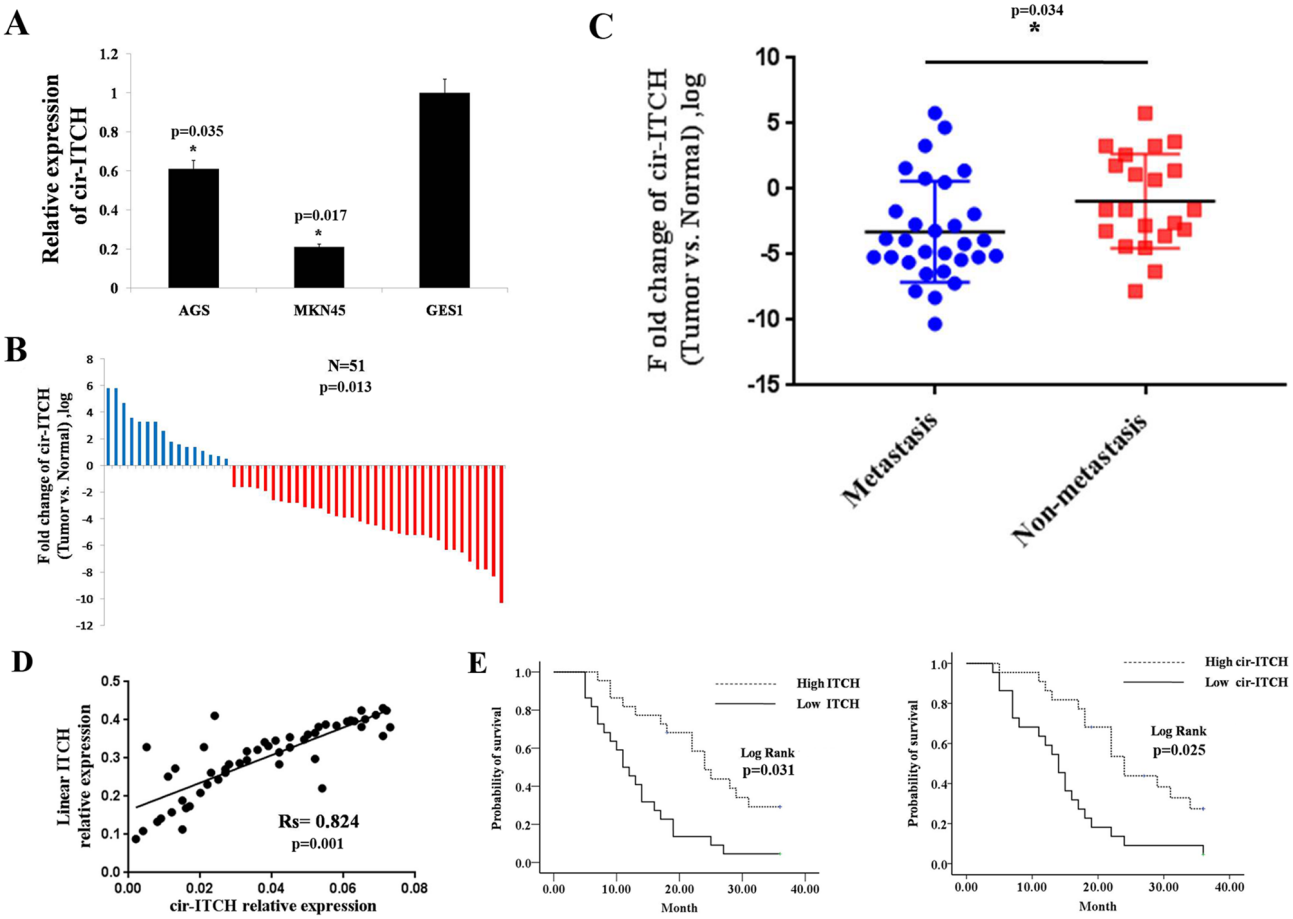
Cir-ITCH expression is downregulated in gastric cancer tissues and cell lines. (**A**) Cir-ITCH expression was downregulated in gastric cancer lines (AGS and MKN45) after normalizing to a normal gastric mucosa cell line (GES1). The data are presented as the means ± s.d. (n = 3) for the cell lines *, *p* < 0.05. (**B**) Cir-ITCH expression was significantly increased in gastric adjacent normal mucosa samples. (**C**) Cir-ITCH expression levels were downregulated in metastatic gastric cancer samples. The data are presented separately for human samples; * *p* < 0.05. (**D**) Cir-ITCH expression was positively correlated with linear ITCH expression in gastric cancer. (**E**) Kaplan–Meier survival analysis showed that patients with low cir-ITCH or ITCH levels had significantly shorter overall survival. The data are presented separately for human samples; * *p* < 0.05.

First, we examined cir-ITCH expression in gastric cell lines using TaqMan-based RT-qPCR assays and observed that cir-ITCH expression was decreased in the gastric cancer cell lines (AGS and MKN45) compared to that observed in normal gastric mucosa cells (GES1) (Fig. 2A). To further investigate cir-ITCH expression in gastric cancer, we examined cir-ITCH expression in human gastric cancer samples and paired adjacent normal mucosa samples by TaqMan-based RT-qPCR assays. We observed that cir-ITCH expression was higher in the adjacent normal mucosa compared to that observed in matched gastric cancer samples (Fig. 2B). We next assessed the relationship between cir-ITCH and clinical parameters (Table 3) and observed that cir-ITCH expression levels were significantly higher in patients without metastasis than in those with metastasis (Fig. 2C).

**Table 3:** Clinicopathological characteristics of the patient cohort.

Characteristics	Number of patients	Average folds.log	*P* values
		(tumor vs. Normal)	
**Age **(**years**)			
≤ 60	23	− 2.27	0.966
> 60	28	− 2.32	
**Gender**			
Male	34	− 2.99	0.073
Female	17	− 0.91	
**Smoke**			
Yes	21	− 1.51	0.235
No	30	− 1.84	
**Drinking history**			
No	22	− 1.6	0.14
Yes	29	− 3.22	
**HP infection**			
Yes	36	− 2.68	0.284
No	15	− 1.38	
**Pathological T**			
PT0-T2	26	− 2.05	0.65
PT3-T4	25	− 2.55	
**Tumor differentiation**			
Poorly	27	− 2.84	0.298
High/Middle	24	− 1.69	
**Lymph node metastasis**			
N0	21	− 0.92	0.034*
N1-3	30	− 3.26	

*p < 0.05.

### Prognostic significance of cir-ITCH and linear ITCH in gastric cancer

We subsequently assessed linear ITCH expression in 51 adjacent normal mucosal tissues and gastric cancer tissues by RT-qPCR. The results showed that patients with higher cir-ITCH expression levels in gastric cancer tissues exhibited substantial upregulation of linear ITCH (Fig. 2D). To determine whether the overall survival of gastric cancer patients was correlated with cir-ITCH, a K-M survival analysis was performed. We divided the patients into different groups, including low and high cir-ITCH groups and low and high linear ITCH groups (using median values as the cutoff). The high cir-ITCH and linear ITCH groups had significantly higher overall survival time than the low cir-ITCH and linear ITCH groups (Fig. 2E).

### Interaction between cir-ITCH and miRNA

**Figure 3:**
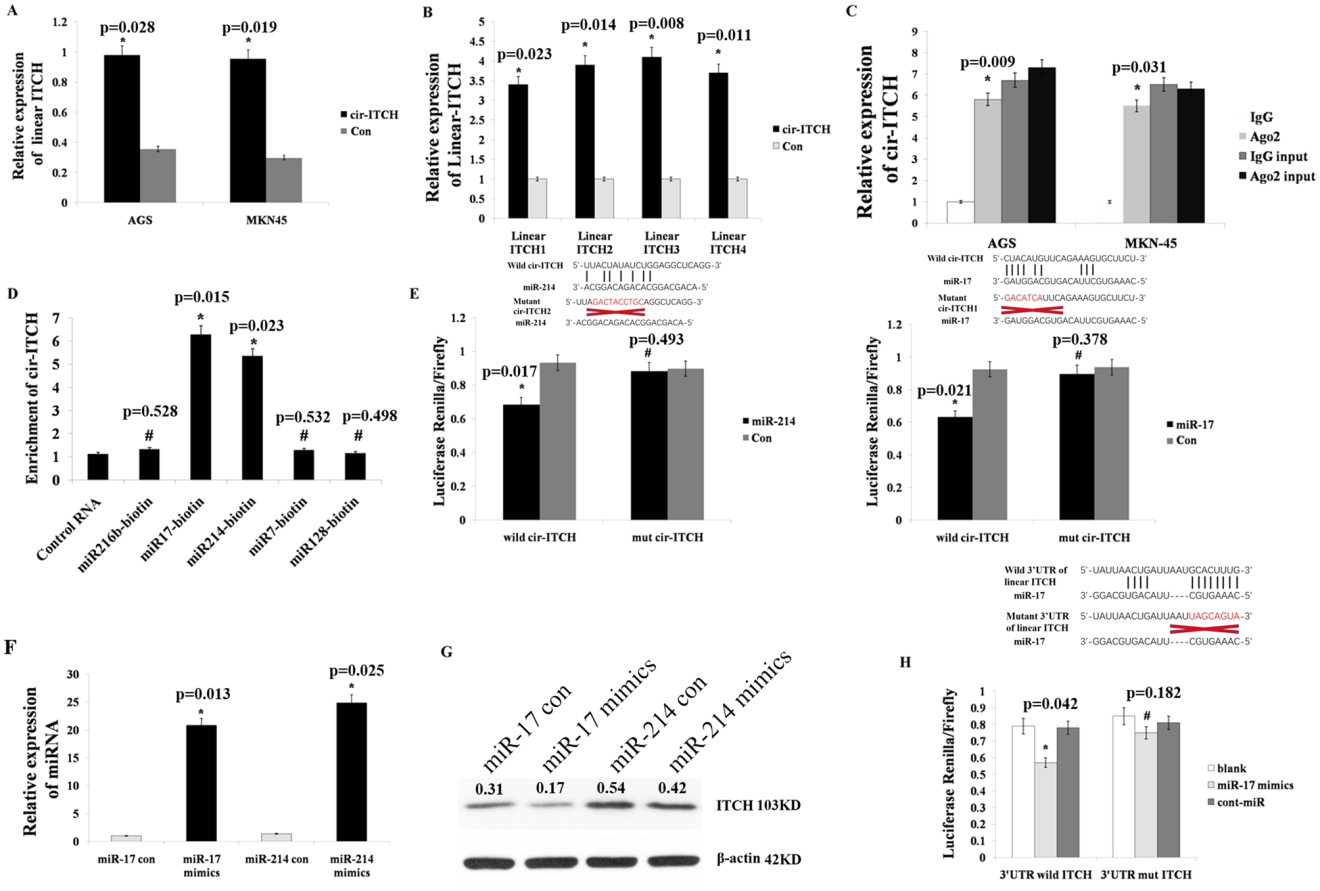
Cir-ITCH increases linear ITCH expression by directly binding to miR-17. (**A**) RT-qPCR results proved that cir-ITCH can increase linear ITCH expression in the gastric cancer cell lines AGS and MKN45. (**B**) Linear ITCH that did not originate from the cir-ITCH exon showed significantly increased expression after cells were transfected with cir-ITCH. (**C**) RIP assay results showed that cir-ITCH could be pulled down by anti-Ago2 in AGS and MKN45 cells. (**D**) The pull-down assay results showed that only miR-17/miR-214 caused a fold change in cir-ITCH in the complex, whereas miR-216 b, miR-7 and miR-128 did not. (**E**) Top panel: Wild-type cir-ITCH but not mutant cir-ITCH could sponge miR-17 and miR-214. Lower panel: miR-17 and miR-214 significantly inhibited the luciferase activity of the wild-type circRNA-ITCH reporter but not that of the reporter vector containing mutated binding sites in circRNA-ITCH. (**F**) miR-17 and miR-214 expression was significantly enriched after cells were transfected with miRNA mimics. (**G**) MiR-17 but not miR-214 overexpression could significantly attenuate ITCH protein expression. (**H**) Top panel: miR-17 could bind to wild type 3′UTR of linear ITCH but miR-17 could not bind to mutant 3′UTR of linear ITCH. Lower panel: Luciferase reporter assay results showing that miR-17 mimics could significantly decrease the luciferase activity of the wild-type but not the mutant linear ITCH reporter. The data are presented as the means ± s.d. (n = 3) for the cell lines *, *p* < 0.05, #, *p* > 0.05.

To further analyse the relationship between cir-ITCH and linear ITCH, we examined linear ITCH expression in AGS and MKN45 cells harbouring cir-ITCH-overexpressing or empty vectors. Linear ITCH expression was significantly increased in the cir-ITCH-overexpressing gastric cell lines (Fig. 3A). To rule out the possibility that the elevated linear ITCH levels originated from the artificial cir-ITCH vector, we redesigned different primers far away from the cir-ITCH exon. Using these primers, we examined linear ITCH expression and observed that it was also significantly increased in cells harbouring the cir-ITCH-overexpressing vector (Fig. 3B). These results demonstrated that cir-ITCH could elevate linear ITCH expression via an unknown pathway. As the primary role of circular RNAs is to sequester miRNAs by sponging them, we postulated that cir-ITCH can sponge some unique miRNAs that can also bind to the 3′UTR of linear ITCH. Using four prediction tools (Miranda, PicTar, TargetScan and miRDB), we predicted that miR-216 b, miR-17, miR-214, miR-7, and miR-128 could simultaneously bind to the 3′UTR of cir-ITCH and ITCH. Previous studies have shown that Ago2 can bind to both circRNAs and miRNAs^26,27^. To determine whether cir-ITCH can sponge miRNA, we performed an Ago2 RIP assay to pull down RNA transcripts that bound to Ago2 in AGS and MKN45 cells. The results showed that endogenous cir-ITCH was efficiently pulled down by anti-Ago2 (Fig. 3C). To further assess whether cir-ITCH could sequester miRNA, a biotin-coupled miRNA capture assay was performed. Interestingly, cir-ITCH was only efficiently enriched by miR-214 and miR-17 and not by the other three miRNAs (Fig. 3D). In addition, we also used luciferase reporter assays to investigate whether miR-214 and miR-17 directly interact with cir-ITCH. Luciferase expression significantly decreased after cells were transfected with miR-214 or miR-17 and wild-type cir-ITCH. However, luciferase expression was unaltered after the cotransfection of miR-214 or miR-17 with mutant cir-ITCH (Fig. 3E). These results show that cir-ITCH can act as a molecular sponge for miR-214 and miR-17. To assess whether miR-214 and miR-17 can affect linear ITCH expression, we increased miR-214 and miR-17 expression using miRNA mimics and their expression was significantly elevated in the mimics group compared to that observed in the control group (Fig. 3F). Then, we assessed ITCH expression and observed that linear ITCH expression was significantly disrupted by miR-17 overexpression. Interestingly, ITCH expression was not inhibited by miR-214 overexpression (Fig. 3G). To rule out an indirect interaction between linear ITCH and miR-17, we performed luciferase assays. We constructed wild-type and mutant linear ITCH 3′UTR fragments, where the miR-17-binding site was mutated in the latter construct by site-directed gene mutagenesis kit. Then, we inserted these fragments into the plasmid pmirGLO. After transfecting AGS cells with miR-17 mimics and pmirGLO plasmid constructs, we performed luciferase reporter assays and observed that miR-17 mimics could significantly decrease luciferase activity for the wild-type ITCH construct compared to that observed in the mutant ITCH and blank groups (Fig. 3H). These results indicated that cir-ITCH directly inhibits linear ITCH expression via miR-17.

### Cir-ITCH attenuates gastric cancer cell proliferation, migration and invasion by sequestering miR-17

**Figure 4:**
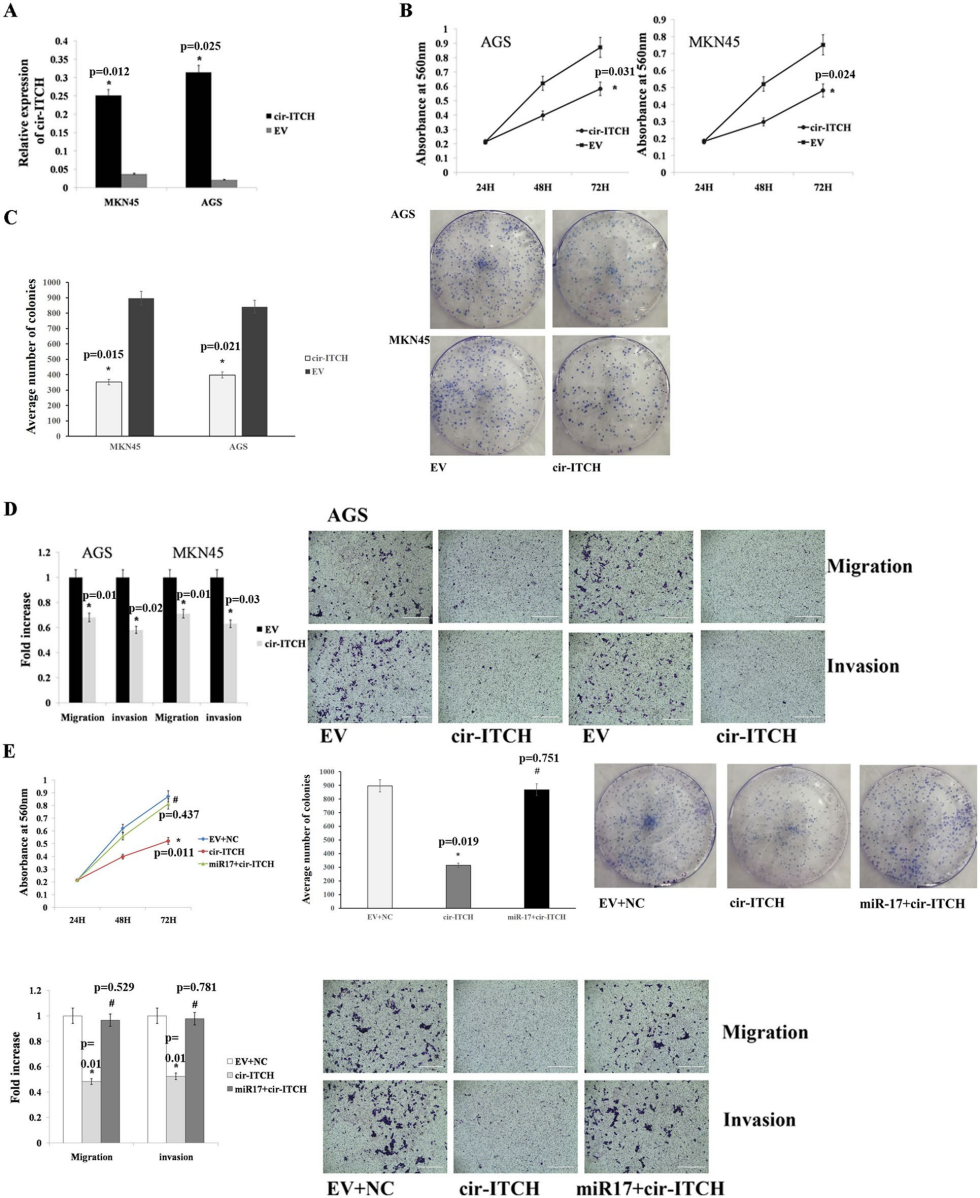
Cir-ITCH inhibits gastric cancer cell migration, invasion and proliferation by sequestering miR-17. (**A**) RT-qPCR results showed that cir-ITCH expression was increased in AGS and MKN45 cells transfected with the cir-ITCH overexpression vector. (B-C) Colony formation and CCK-8 assay results showed that cir-ITCH overexpression significantly reduced the proliferation and colony formation of AGS and MKN45 cells compared to that observed in the control. (**D**) Transwell migration and Matrigel invasion assay results demonstrated that cir-ITCH significantly decreased the migration and invasion of AGS and MKN45 cells compared to that observed in the control. (**E**) MiR-17 overexpression restored the tumourigenic qualities of AGS cells compared to that observed in the control. The data are presented as the means ± s.d. (n = 3) for the cell lines *, *p* < 0.05.

To study the function of cir-ITCH in gastric cancer, the cir-ITCH overexpression plasmid was transfected into MKN45 and AGS cells, and we observed that cir-ITCH expression was significantly increased in both cell lines (Fig. 4A). We then performed CCK-8 and colony formation assays to evaluate the effects of cir-ITCH on gastric cancer cell proliferation. The proliferation of AGS and MKN45 cells was significantly reduced in the cir-ITCH overexpression group compared to that observed in the control group (Fig. 4B). The cir-ITCH-overexpressing cells also exhibited reduced colony formation abilities, fewer foci were observed in cir-ITCH-overexpressing cells than in the control cells (Fig. 4C). In addition, we observed that cir-ITCH could significantly reduce the migration and invasion of gastric cancer cells (Fig. 4D). To further verify whether cir-ITCH can inhibit gastric cancer proliferation, migration and invasion by sponging miRNAs, we performed rescue experiments. The results showed that miR-17 restoration could recover gastric cancer cell migration, invasion and proliferation in the AGS cell line (Fig. 4E). Taken together, these results showed that cir-ITCH can attenuate gastric cancer cell proliferation, migration and invasion by sequestering miR-17.

### Cir-ITCH decreases tumour growth by sequestering mir-17 in vivo

**Figure 5:**
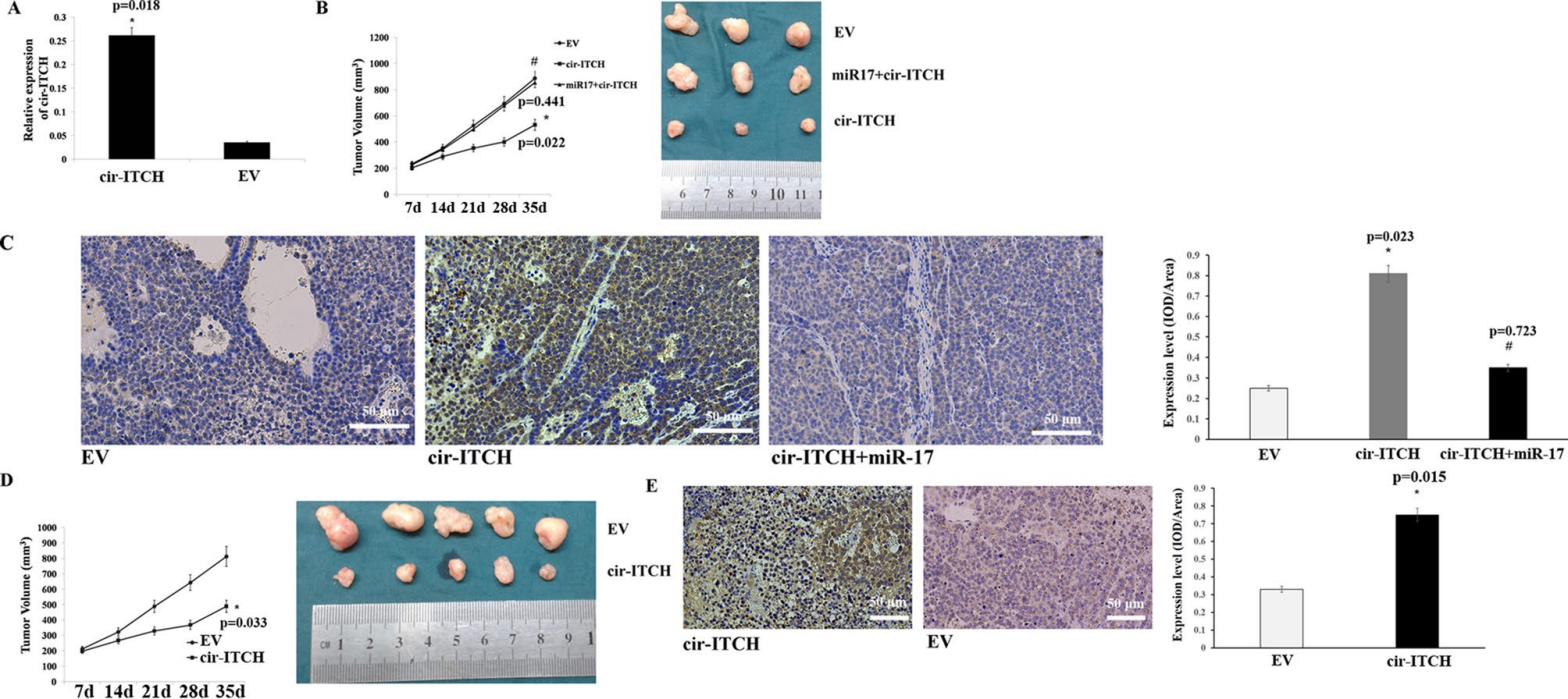
Cir-ITCH inhibits gastric cancer growth via miR-17 in nude mice. (**A**) Cir-ITCH expression was significantly increased in tumours formed by cells transfected with the cir-ITCH vector compared to those formed by cells transfected with the empty vector. (**B**) Left panel: Primary tumour growth after orthotopic injection of AGS cells overexpressing cir-ITCH or harbouring the EV with or without miR-17 restoration. Right panel: Image of xenograft tumours from nude mice. The xenograft tumour models showed that tumours grown from cir-ITCH-overexpressing cells were smaller than those grown from control cells. The data are presented as the means ± s.d. (n = 3) *, *p* < 0.05. (**C**) Left panel: ITCH expression was detected by immunohistochemical staining in the control group, cir-ITCH overexpression group and cir-ITCH and miR-17 group. Right panel: ITCH expression was quantified using the IOD/Area method. (**D**) Left panel: PDTX growth after intratumoural injection with the cir-ITCH vector or empty vector. Right panel: Image of PDTXs. The PDTX models showed that intratumoural injection of cir-ITCH significantly inhibited tumour growth. The data are presented as the means ± s.d. (n = 5) *, *p* < 0.05. (**E**) ITCH expression in the control and cir-ITCH overexpression groups was detected by immunohistochemical staining.

In vitro, cir-ITCH was shown to significantly reduce the tumourigenesis of gastric cancer. To further investigate the function of cir-ITCH in vivo, we performed the following experiments. First, we observed that the expression of cir-ITCH was significantly increased in tumour xenografts overexpressing cir-ITCH (Fig. 5A) Then, we performed the rescue experiment and observed that cir-ITCH overexpression significantly inhibited tumour growth in vivo, whereas miR-17 overexpression restored tumour growth (Fig. 5B). Then, we assessed ITCH expression in tumour xenografts through immunohistochemical analysis. We observed that ITCH expression was significantly increased in the cir-ITCH overexpression group, and ITCH expression was restored after simultaneously overexpressing miR-17 and cir-ITCH (Fig. 5C).

Because patient-derived tumour xenografts (PDTXs) can retain the human tumour microenvironment and maintain interactions between the innate immune system and tumour cells, PDTXs are considered a translational model for cancer studies^28^. Therefore, we designed a PDTX model from a male gastric cancer patient and assessed the therapeutic effect of cir-ITCH by intratumoural injection of cir-ITCH or an empty vector. The injection of cir-ITCH significantly attenuated PDTX growth in vivo (Fig. 5D), while ITCH expression significantly increased (Fig. 5E). These results indicate that cir-ITCH is a promising therapeutic target in gastric cancer.

### Cir-ITCH is involved in regulating the Wnt/β-catenin signalling pathway

**Figure 6:**
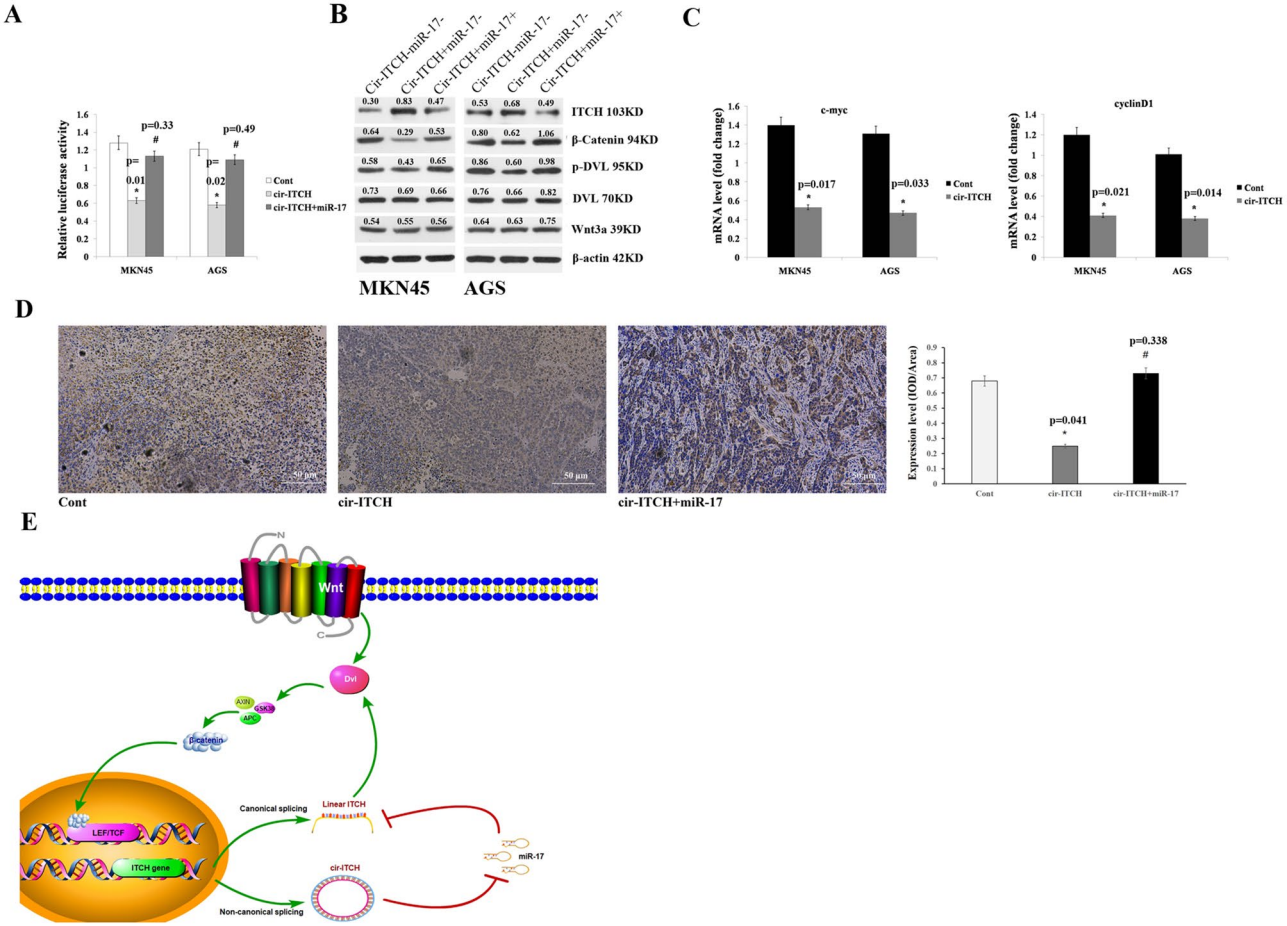
Cir-ITCH is involved in regulating the Wnt/b-catenin signalling pathway. (**A**) TCF luciferase reporter assay results showed that cir-ITCH overexpression significantly inhibited TOPflash activity compared with that observed in the control group, while miR-17 could restore TOPflash activity. (**B**) The protein levels of ITCH, β-catenin, Wnt3a, Dvl, and p-Dvl were assessed in AGS and MKN45 cells by western blot analysis. Cir-ITCH overexpression significantly inhibited β-catenin and p-Dvl levels compared with that observed in the control group, which could be restored by miR-17. (**C**) The mRNA levels of c-Myc and cyclin D1 were detected by RT-qPCR after cells were transfected with cir-ITCH or empty vector. Cir-ITCH overexpression significantly decreased c-Myc and cyclin D1 expression compared with that observed in the control group. The data are presented as the means ± s.d. (n = 3) *, *p* < 0.05. (**D**) Left panel: β-Catenin expression was assessed in the control group, cir-ITCH overexpression group and cir-ITCH and miR-17 overexpression group. Right panel: β-Catenin expression was quantified using the IOD/Area method. (**E**) Schematic representation of the cirITCH-miR17-linear ITCH-Dvl axis.

Wei W et al. showed that ITCH plays a negative regulatory role in modulating canonical Wnt signalling by targeting the phosphorylated form of Dvl2^29^. As previous results indicated that cir-ITCH can regulate linear ITCH expression by sponging miR-17, we postulated that cir-ITCH could also modulate canonical Wnt signalling. Therefore, we performed a β-catenin/TCF-responsive luciferase reporter assay to assess if cir-ITCH can regulate the Wnt/β-catenin signalling pathway in gastric cancer. In addition, we observed that cir-ITCH overexpression could significantly inhibit TOPflash activity, while miR-17 overexpression could restore TOP flash activity (Fig. 6A). In addition, ITCH, β-catenin, Wnt3a, Dvl2, p-Dvl2 levels were analysed by western blot assays in AGS and MKN45 cells with cir-ITCH hyperexpression. The results showed that β-catenin and p-Dvl2 expression was significantly decreased in the cir-ITCH hyperexpression group, whereas no change in Wnt3a expression was observed. Similar to previous results, miR-17 overexpression could also restore β-catenin and p-Dvl2 expression (Fig. 6B). Furthermore, we noted that cir-ITCH could significantly suppress endogenous Wnt target genes, such as c-Myc and cyclin D1, in AGS cells through qRT-PCR analysis (Fig. 6C). In vivo experimental results also proved that β-catenin expression in xenograft tumours could be suppressed by cir-ITCH, while miR-17 could restore β-catenin expression (Fig. 6D). Based on our results, we can conclude that cir-ITCH can increase linear ITCH expression by sponging miR-17, while linear ITCH can inhibit the Wnt/β-catenin pathway by targeting Dvl (Fig. 6E).

## Discussion

A recent study showed that the non-canonical Wnt signalling pathway contributes to gastric cancer progression via dishevelled (Dvl)^30^, and another study showed that phosphorylated Dvl can be degraded by ITCH via the proteasome pathway^31^. The results of these studies indicate that ITCH may be associated with gastric cancer progression. As cir-ITCH shares some miRNA binding sites with the 3′UTR of ITCH and can regulate linear ITCH expression in different cancers^16–18^, we inferred that cir-ITCH may be a crucial gene in the development of gastric cancer. Therefore, in our present study, we investigated the expression and function of cir-ITCH in gastric cancer. First, cir-ITCH expression was observed to be decreased in gastric cancer tissues and cell lines. Furthermore, cir-ITCH expression was correlated with tumour metastasis. These results showed that cir-ITCH, as a tumour suppressor gene, was expressed at lower levels in gastric cancer and was associated with metastasis. To further study the relationship between linear ITCH and cir-ITCH, we assessed linear ITCH expression in gastric cancer tissues. As expected, we observed that cir-ITCH expression was positively correlated with that of linear ITCH. Our results also showed that gastric cancer patients with high levels of linear ITCH and cir-ITCH have long overall survival. Therefore, linear ITCH and cir-ITCH may serve as prognostic markers for gastric cancer patients.

Our results indicated that cir-ITCH overexpression could upregulate linear ITCH expression, but the mechanism remained unclear. As circRNAs can sequester the miRNAs^32,33^, we speculated that cir-ITCH may increase linear ITCH expression by sponging some miRNAs. The results of Ago2 RIP assays proved that cir-ITCH could bind to a number of miRNAs in gastric cancer, but which miRNAs were bound by cir-ITCH remained unknown. We identified five miRNAs using prediction tools and subsequently observed that cir-ITCH was only efficiently enriched by miR-214 and miR-17 using biotin-coupled miRNA capture assays. These results indicated that cir-ITCH can sponge miR-214 and miR-17 in gastric cancer. Subsequent luciferase reporter and actinomycin D assays also proved that cir-ITCH could directly bind to miR-214 and miR-17.

Many studies have shown that miR-214 and miR-17, as oncogenes, may promote gastric cancer migration, invasion and proliferation. For example, Chen et al.^34^ showed that miR-17-5p promotes gastric cancer proliferation, migration and invasion by directly targeting early growth responses. Qu et al.^35^ demonstrated that mR-17-5p regulates cell proliferation and migration by targeting transforming growth factor-β receptor 2 in gastric cancer, and Yang et al.^36^ showed that miR-214 promotes gastric cancer cell proliferation, migration and invasion by targeting PTEN. Indeed, our results showed that cir-ITCH could inhibit gastric cancer tumourigenesis by promoting linear ITCH expression through miR-17 in vivo and vitro. Unfortunately, the possible mechanism regarding how ITCH affects gastric cancer tumourigenesis remains unclear.

The results of some studies have indicated that phosphorylated Dvl2 can be degraded and ubiquitinated by ITCH^29^, leading to the inhibition of canonical Wnt signalling. As the Wnt/β-catenin pathway plays a crucial role in many cancers, including stomach tumour^37^, hepatocellular carcinoma^38^, and pancreatic cancer^39^, we postulated that elevated ITCH levels can inhibit Wnt signalling in gastric cancer and lead decreased tumourigenesis. To test our hypothesis, a β-catenin/TCF-responsive luciferase reporter assay was performed to examine whether cir-ITCH can regulate the Wnt/β-catenin signalling pathway. The results showed that cir-ITCH overexpression significantly inhibited TOPflash activity and downregulated β-catenin and p-Dvl expression, while miR-17 could completely counteract these effects. In addition, the expression of endogenous Wnt target genes such as c-Myc and cyclin D1 was suppressed by cir-ITCH overexpression. These data demonstrated that cir-ITCH has an inhibitory effect on the canonical Wnt pathway.

In summary, our results showed that cir-ITCH is a prognostic marker that is downregulated in gastric cancer and that cir-ITCH can prevent gastric cancer tumourigenesis by sequestering miR-17 through the Wnt/β-catenin pathway.

## Supplementary information

Supplementary file1
