# Supplementary material for: Cir-ITCH inhibits gastric cancer migration, invasion and proliferation by regulating the Wnt/β-catenin pathway
Source: Sci Rep. 2020 Oct 15;10:17443. doi: 10.1038/s41598-020-74452-8 (PMC7566509; doi:10.1038/s41598-020-74452-8)
Supplement: Supplementary file 1 — Supplementary file1 [file 41598_2020_74452_MOESM1_ESM.docx]

**Cir-ITCH Inhibits Gastric Cancer Migration, Invasion and Proliferation by Regulating the Wnt/β-Catenin Pathway**

**Yang Peng ^1^, Hong Hong Wang^2^***

^1^Department of Geriatrics, Tongji Hospital, Tongji Medical College, Huazhong University of Science and Technology, Wuhan 430030, China

^2^Department of Ultrasonography, Tongji Hospital, Tongji Medical College, Huazhong University of Science and Technology, Wuhan 430030, China

*Author to whom correspondence should be addressed. E-Mail: 85honghong@163.com

Corresponding Author:

Full name: Hong Hong Wang

Institute: Tongji Hospital, Tongji Medical College

Department: Department of Ultrasonography

University: Huazhong University of Science and Technology

Street Name & Number: 1095# Jiefang Avenue

City, State, Postal code, Country: Wuhan 430030, China

Tel:+8615927451391

E-mail:85honghong@163.com
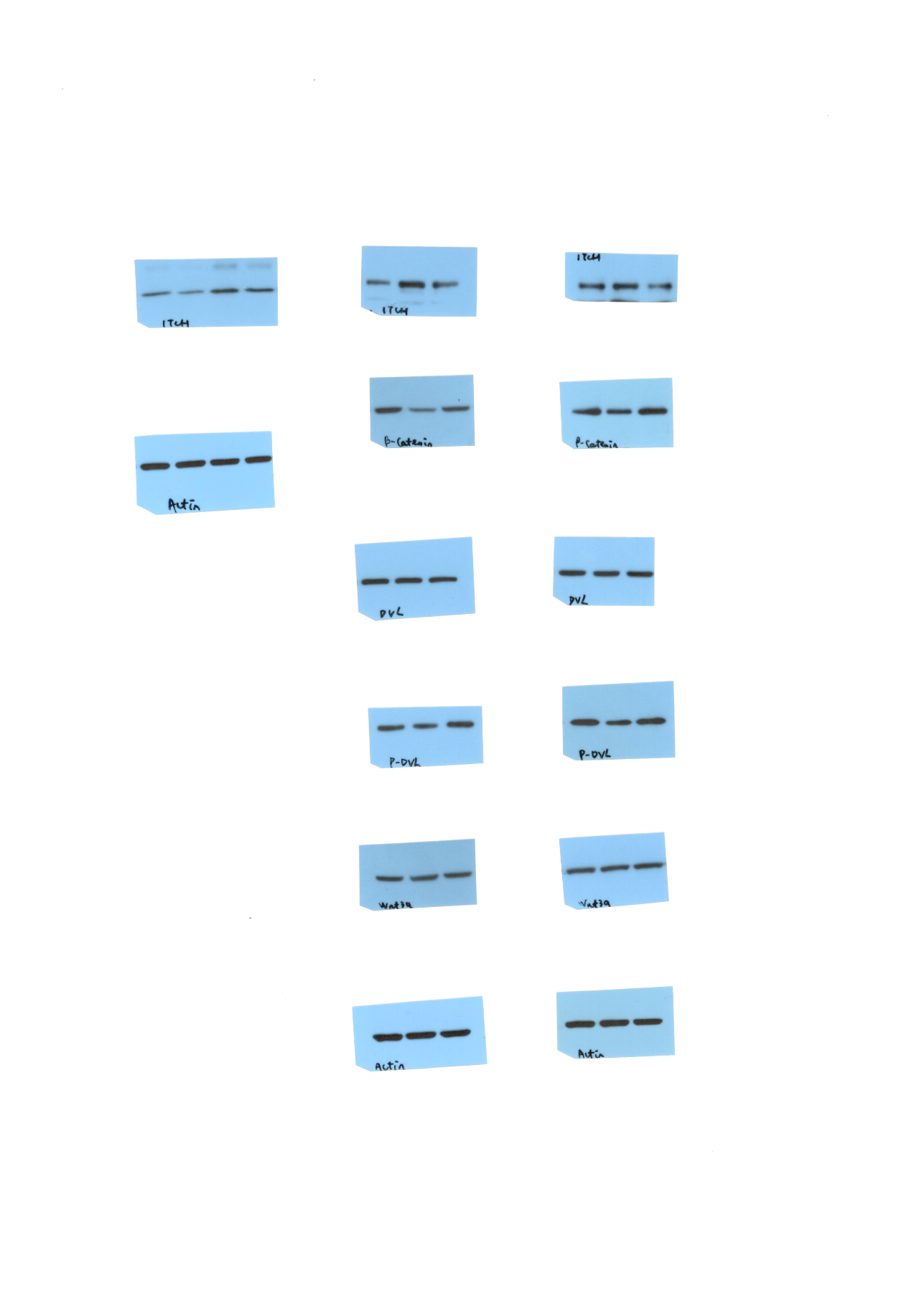


**Original western blot figure**
